# Editors’ Table

**Published:** 1857-01

**Authors:** 


### Editors' Table.

The opening of a new year, always marked by an interchange of friendly greeting, and by budding hopes of future advantages and distinction, is not without its influence on ourselves. To our readers we offer more than the expression of our good wishes for their welfare and the maturity of their hopes, —we offer them a new Journal, fraught with the productions of many minds, each bearing evidence of careful and conscientious elaboration. In exchange, we hope to receive a frank approval of our efforts on behalf of all those to whom we now address ourselves, and a due measure of support at their hands. They can aid us in different ways—by their names, and the exercise of their pens for the promotion, not of our cause alone, nor of theirs alone, but of the common cause. We volunteer to lead the way, but with no desire or ambition to be at the head of a forlorn hope. We would rather be joined by a glad array of those who, on becoming members of the profession, virtually enlisted to serve under the same banner with ourselves, for the protection of the weak, the preservation of the strong, and the driving out of diseases from their many fastnesses.

Happily, favoring circumstances allow us to believe that we shall be thus supported in our onward course. The union of the Louisville Review with the Philadelphia Medical Examiner gives encouragement and promise of strength, which it will be our earnest endeavor to realize, at least to some extent. Confiding in a continuation of the goodwill of the friends who have sustained us from the beginning in the Review, are we too sanguine in our speaking in advance of our new constituents who were formerly those of the Examiner, and in counting upon their adhesion to its chosen successor and a new ally under a different patronymic? The industry, good taste, sound ethics, punctuality, and gentlemanly bearing of the late editor, Dr. S. W. Hollingsworth, will not be lost on the conductors of the North American Medico-Chirurgical Review; and although his retirement from the editorial corps deprives us of his aid in this way, we shall foster the hope that he will, as occasion may call forth, give us the benefit of his practical knowledge and medical lore.

For ourselves, we are fully sensible of the heavy responsibility incident to our present position; but we derive encouragement from the reflection, that neither change of place nor the expectation of a reinforcement of new friends, require any change in our principles of action, or any modified programme with a view of propitiating a particular interest or party. If our passwords are now no longer Louisville or Philadelphia, as applied to the Review and the Examiner, we shall have none the less regard for the honor and elevated standing of the profession in these cities than before; and while our love for the South and West is as ardent as ever, our regard for the East will be not less strong than it is in those "to the manor born." We do not mean that our course shall be marked by any civic or sectional bias, nor, *a fortiori*, by personal predilections, and still less by personal prejudices, if we should ever be so unfortunate to feel them. We make our appeal to the whole country, on the expectation that it will be responded to in such a manner as to justify the title of our Journal, and to make it the vehicle of sound doctrines and available practice. And in this connection we beg leave to say a few words in answer to the question:

### WHAT IS THE PROPER SPHERE OF EDITORIAL LABOR?

We propose no homily on the duties and responsibilities of editorial life; but we would like, for a moment, to glance over the field which we propose to occupy.

Aside from the discussion of matters purely scientific, the medical periodical has lately found, in the profession itself, the source of an occasional and profitable creed of doctrine. In spite of the woful Jeremiads so unceasingly chanted in our ears about the spread of quackery, and the decline of legitimate medicine in the affections of the American public, we are inclined to take a cheerful view of the present position of the profession in this country. No ululation to the contrary can convince us that it lacks in healthful growth and energy. Amid all the untrammelled license, the deep-rooted scepticism born of free institutions, our claim to respectable antiquity, and to a veneration based on what our fathers have done, goes for nothing; and yet, under such circumstances, only three-fourths of a century have sufficed to build up a profession, united, strong, energetic, having already a glorious history, having before it a still more glorious future. Out of our poverty in government patronage and support, our colonial position, our want of great institutions of learning, our expensive system of medical teaching, we have builded up an honest claim on the gratitude of posterity, and the support of the present generation.

The fellowship of the profession should be its chiefest pride, as it is its strongest bulwark. The rapid westward stride of civilization builds up new states in a day, but before the haste of politicians or the rush of emigration can frame a constitution, the physicians are there, have met and organized a society, and find willing entrance for their delegates in our national medical congress. Unprotected by legislation, we find a remedy and a substitute in those affiliations which band us together in a common brotherhood.

The power of this associated effort should not be lightly estimated, and who shall say that in its guidance the press shall have no influence? That is a mistaken sentiment which would exclude medical policy from our medical journals; which would confine them to scientific discussion, and leave our vast and complicated organization to work on, unwatched and uncared for. The success and respectability of the profession, its very attainments in learning, depend, to no small extent, upon the continued prosperity of our city, county, state, and national societies. They are our strongholds, and whatever weakens them, strikes at the heart of our success. Imagine for a moment that all these organizations were disbanded, and the profession turned loose upon the world, each man to fight his own battles, with only his own sense of propriety for his guide. Should we not regret the loss of that feeling of fellowship with which the physician of Maine now takes the hand of one of California? Should we not feel the want of control over those restless or dishonorable spirits, which a body so large as ours must necessarily have among its members?

We need not multiply reasons. These organizations, which we so highly prize, must be fostered and cared for—their purity of purpose must be preserved, they must not become subservient to local interest, their honors must be conferred only on the worthy—those who have earned them by well-doing—and they must seek out new channels of influence, and be made the nursing-mothers of science.

Unfortunately, our profession, so noble in the aggregate, is made up of human hearts. The personal ambitions, the hatreds and jealousies of active competition, the favoritism of cliques, will creep into ours as into all other organizations; and it is one of the proudest features of the profession, that so little evidence of the lower passions of humanity has shown itself, as yet, in them. Thus far, their history is grand—not faultless, not without matters for regret—but still wonderful in their purity, when the paths of chicanery have been left so open.

To maintain and extend this purity is one of our dearest hopes. We would not look harshly on the motives which have in some instances led to its violation. But we do need a firm, calm censor, one who has strength and courage to speak out when a word spoken in season may work for good; we need the guiding power, the open discussion of the press, to enlighten professional sentiment.

Let us be understood. We detest the "fearless editor," *par excellence;* that truculent fellow who announces that his mission is reform, that an Augean stable is to be cleansed, and that he is the Herculean scavenger whose arm shall do it; who labors under the impression that somebody should be held up to public scorn and indignation in every issue; and that the best way to correct an evil is by irritating abuse of every one who has the power to correct it. But if this is not our policy, we are equally at variance with that feeble selfishness which has no voice, that facile soul which has no principles. We would see the medical journalist bold and manly; but we would have him recollect that an evil is an evil in and of itself, and that, in any attempt to reform it, the fewer personal feelings involved, the greater the chance of success. The old catechetical distinction between the sin and the sinner was founded on good, strong, Christian common sense and shrewdness. There is a deep philosophy of human nature in its teachings.

Our colleges, those noble institutions, built up, not from the wealth of the state, as pensions and sinecures for lazy favorites, but out of their own necessity, and the inevitable pecuniary prosperity of that which is needed, are, with all their excellencies, a little too independent for the safety of the profession. They acknowledge no control but that of professional sentiment. How shall this find expression? How may public opinion be brought to bear upon them, to keep them off the track of unlicensed peddling of diplomas; and, stimulating their competitions, force them to elevate higher and higher their standard for the doctorate? Only through the press. It speaks with the voice of a thousand men; and no college in the land is strong enough to withstand its censure, if that censure be just and calm.

Our system of education is the natural outgrowth of our national character. Free, voluntary, and strong in its individuality, it is the one best adapted to the American mind. Each school relying on its merits for its existence, an active competition is engendered, which, healthful in itself, is liable to excesses. A check here, or encouragement there, is needed; above all, the system itself should be preserved. All our usefulness, our proud reputation abroad, has grown from it; and we should be cautious how we permit the innovations of a too rapid democracy, or substitute for it the methods of the transatlantic world. Our literature, the weakest point in our national character, is another field for editorial labor. The detail of criticism belongs to our department of reviews, but the broad, general principles which should govern criticism itself, belong here in our editorial pages. We risk nothing in the assertion that the profession of the United States is better educated than that of France; but can we, with any propriety, claim that we approach them in literary merit? The learning of our country is diffused; there is a better average and a better aggregate here than in any other; but alas, the teachers, the slaves of the lamp, are wanting. We have but few names with which we can cross the Atlantic, and say: "These are our stars—show us brighter if you can!"

But we have a few. How shall we multiply them, how temper down the passion for notoriety, and instil in its stead a love for well-earned, honest fame? Shall we, in our love for American literature, our ardent desire to see it usurp the place of European reprints in our libraries, and our disgust at our colonial position, take to our arms every maker of a book, and write his Avatar under the name of a review? Shall we pet compilers, endorse stupidity, and cackle in triumph over the soft-shelled eggs of unfledged aspirants to literary honors?

On the other hand, shall we systematically depreciate the American author? Shall we write "Nazareth" over the American coat of arms, and say, "No good can come from hence?'

Much can be gained, not only by sound, intelligent, and honest reviews of new books, but by the establishment of some definite policy with regard to their encouragement. American writers, and publishers as well, require fair treatment and a liberal policy.

But who shall guard the shepherds? What necessity for new journals, when our exchange list numbers some two score, all published within the broad area of our country? Journalism itself is open to improvement. Few of our periodicals are rich enough to be independent. They are born most commonly, not because they are needed, but because their existence is a necessity to some school or some clique. Their multiplication is an evil; not but that the poorest has its sphere of usefulness, but because they occupy the attention of the profession, and distract it from better publications. We shall not be accused of murderous designs upon the existence of our contemporaries, or of jealousy of their success, when we declare our opinion that the medical mind is too much diffused through the numerous channels of intelligence. One or two good writers cannot make a good journal; the learning, talent, and industry of many minds should be concentrated, in order to bring about the highest excellence. When this is done we shall have fewer medical periodicals, but better ones. And what an influence would such a literature exert upon the progress of medicine where progress is most needed, in the minds of practitioners in city and country.

Already our numerous monthlies are written above the capacity of many of their readers. Their mission of education is as yet unfulfilled, and hence we are inclined to believe that the time for a reformation, by reducing their number, has not yet arrived. Perhaps it may never come; but even should the present system of scattered effort continue, progress and improvement are inevitable. After all, it is not the man of learning and cultivation, the man with an overflowing library, polyglot in its languages, all of which are mother tongues to him, who needs the efforts of the teacher. It is the man in the rural districts, doing his best on a small income, without a library other than the text-books of his student-life, without languages other than the homely English learned at the common school, who calls for instruction. To adapt a periodical to these varied purposes, to furnish a means of intercourse for cultivated minds, and at the same time elevate the masses, is the most difficult, but most essential duty of the journalist.

It can be done. The ideas of the *true* philosopher are always intelligible, even to uncultivated minds; and, recollecting that, we take courage.

Such is the channel we would mark out for our river of thought, to which are tributary so many earnest minds. It may flow out to this side or to that, cutting here a new sluiceway for opinion, and there abandoning its old bed of error; but it must tend always toward the common reservoir, the ocean of truth.

We trust that we have said enough to vindicate our reasons for considering the polemics of medicine a fair subject for editorial comment. We would have said less, but the open declarations of some influential writers, no less than the established policy of most of our periodicals, have indicated a rigid confinement to scientific discussions as the only proper career for the editor.

We have wished to prove that our professional organizations into societies, our colleges, our literature, standard and ephemeral, constitute the aggregate profession of medicine, as distinguished from the isolated practitioner; that it must have a policy for good or for evil; and that some well-established general principles should govern that policy. To so frame these principles, and so instil them into the medical mind that they shall live and have power as ruling ideas in our community, strong and vigorous because adapted to our national character, the policy of our free institutions, and the tendencies of the people of our country, is the task at which, earnestly and hopefully, we propose to labor.

---

THE PHILADELPHIA MEDICAL EXAMINER AND DR. HOLLINGSWORTH.—With the union of the interests of the Philadelphia Medical Examiner and the Louisville Review, Dr. S. L. Hollingsworth, the conductor of the former journal, retires from the editorial tripod, which he has occupied with so much grace and dignity for the past three years. His connection with the Examiner has made his name familiar to nearly every reading medical man in the country; and we are sure that we express the general sentiment of the profession when we say that the medical periodical literature of the United States has suffered no inconsiderable loss by his retirement. His numerous admirers and personal friends have the consolation, however, of knowing that his withdrawal was entirely a matter of choice and convenience, at a period in the history of the Examiner when its high character was more widely appreciated, its circulation as large, its prosperity greater, and its nutrient supply larger than at any other.

The only exchange journal that has reached us since the announcement of the preceding news is the *Boston Medical and Surgical Journal*, whose kindly feeling toward, and high appreciation of Dr. Hollingsworth and his labors are thus handsomely expressed:

"It is with unfeigned regret that we are obliged to record the retirement of Dr. S. L. Hollingsworth from the editorial post he has occupied for three years with such signal ability. The '*Medical Examiner*' has been a very welcome visitor to our table, and we shall miss its goodly company. We learn from the December number that the journal will be combined with the '*Louisville Review*,' the new title to be '*The North American Medico-Chirurgical Review*.' It is to be issued every alternate month, under the editorial management of Drs. S. D. Gross and T. G. Richardson.

"While it is pleasant to 'welcome the coming guest' in this instance, we feel very unwilling to 'speed the parting' one; for, although wherever he is, and however occupied, we wish him all success, yet we had a strong selfish desire to keep him in the ranks of the editorial army. Every good heart and skilful hand that departs thus unexpectedly is a personal loss. Dr. Hollingsworth has won golden opinions as an editor, and deserves the lasting gratitude of the profession."

---

QUACKERY—SWINDLING.—If despotism has its odious features, liberty has also its weak side, so that they who enjoy its blessings have to submit every now and then to a heavy tax, rather than their charters should be abridged. In no one thing is this so evident as in the toleration of medical quackery by our constituted authorities, whose zealous endeavors to ward off approaching pestilence, and to see that the people have wholesome food of proper weight and measure, are in painful contrast with their supineness and indifference to the continual frauds practised on the community by boastful pretenders to cure all diseases, consumption and cancer not excepted, by their wonderful compounds, which are either inert or poisonous, and are often destructive to those who are silly enough to allow themselves to be duped by such barefaced impostors. There is, in these transactions, for the most part, falsehood from first to last; the title of the article sold is not borne out by its nature or composition; the alleged pathology of the disease to be cured is untrue, and the promise of a certain cure is continually falsified by death. In fact, quackery is nothing less than swindling, and swindling of the worst kind, for the quack not only obtains money under false pretences, but he, at the same time, sells to the victims of his fraud something worse than a damaged article of merchandise. They in good faith receive from his hand a substance or a compound often poisonous, or exceedingly deleterious in some of its constituents, when they think that they are procuring a true panacea, or depurative syrup, or healing balsam, or blood-purifying pills, each one of which preparations shall operate with unerring certainty in restoring them to health, and which the maker and the vendor know will not be productive of any such certain result as is promised.

We are glad to see that in France the government has brought medical quackery within the penal statute under the appropriate head of "swindling"

(*Escroquerie*). In an able article on this subject, by M. Tardieu, in the Annales de Hygiène Publique et de Médecine Légale, for April, 1856, we find notices of fines and imprisonment inflicted on different individuals, because, in the language of the Court of Appeals, when confirming the judgment of an inferior court in one of the cases, the guilty individual "had recourse to fraudulent devices, consisting chiefly in giving out remedies which *he knew* to be inefficacious, and prescriptions incapable of producing the good effects *which he promised;* and that these lying declarations, backed by fraudulent devices, and by means of which he swindled another party out of a portion of his fortune, constitute not only acts of *quackery*, but exhibit also all the characters of the crime of *swindling*." The sentence passed in this case was three months imprisonment, and a fine of three dollars. Another quack was imprisoned for four months. A fellow, calling himself Dr. Rey de Jougler, who carried on operations on a large scale, flooding all France with his millions of circulars, and who was so busy that he was obliged to procure the aid of ten or twelve secretaries, all of whom were represented by his cook "Pierson," was punished by imprisonment for thirteen months, and a fine of twelve hundred dollars. His copartner in iniquity, by the name of Duval, who acted as an apothecary, and made up the medicines, was subjected to a fine of one hundred and twenty dollars. The soi-disant doctor and omni-curer had also to pay three-fifths of the costs, and his associate two-fifths.

---

A bitter but somewhat amusing paper war has been going on for some months past in the medical journals of Great Britain, concerning the manner in which the late Mr. Liston held the knife in the operation of lithotomy. The controversy was commenced in the Lancet of April last by Mr. Fergusson, who stated in a communication to that journal that the wood-cut in Liston's Surgery, in which the operator is represented as holding the bistoury underhanded while passing it along the groove of the staff, misrepresents the distinguished author, who, Mr. Fergusson asserts, invariably held this instrument overhanded, that is with the forefinger resting upon the back of the blade, as in the ordinary method. In confirmation of this fact, Mr. Fergusson stated that he had assisted Mr. Liston in not less than forty operations, and was perfectly familiar with every particular connected with his mode of operating.

In answer to Mr. Fergusson, Mr. Syme of Edinburgh, contends that Mr. Liston did hold the bistoury as represented, and that Mr. Fergusson never assisted Mr. Liston in a single operation of lithotomy. In a subsequent letter to the Lancet, Mr. Fergusson admitted that he did not *assist* Mr. Liston, but was present at the number of his operations mentioned. As well as we can judge from the various communications pro and con that have appeared, we rather think that Mr. Syme is about to get the better of the Queen's surgeon.

It seems to us, however, at this distance, that the British heroes of the catlin and scalpel are hard put to it, when they resort to such straws with which to beat the public ear, and if we could hope to get their attention in the excitement which they have raised, we would respectfully suggest to these gentlemen that they are making themselves very ridiculous. We should like to know what difference it can make concerning a man's reputation as a lithotomist, whether he holds the knife underhanded or overhanded, or whether in his left hand or his right hand, or both hands, so he succeeds in performing the operation with the greatest ease to himself and safety to his patient.

---

The number of students now attending medical lectures in this city is uncommonly large; the three schools each exhibiting an increase upon last winter's class. We are also happy to be able to state that the proportion of young men from the Southern States is greater than usual—a fact at which we are not at all astonished, considering the well-known character for religious, political, and medical conservatism, which this State, and more particularly this city, have always maintained. In saying this, we do not intend to cast any imputation whatever upon cities less highly favored; but no one will deny, that a community in which the religion of the Bible as opposed to the so-called religion of nature, medical orthodoxy as opposed to homœopathy, hydropathy, and all other forms of heresy, and constitutional liberty as opposed to local prejudices, are combined and find their strongest advocates, is not only entitled to the greatest success in its public institutions, but to the admiration and love of the whole nation. If such is Philadelphia—and we honestly think so—she may well treat with scorn and indifference the malignant efforts which are made in certain quarters to tarnish her fair fame. We refer now more particularly to a paragraph which is going the rounds of the medical journals and political newspapers; a paragraph which, we are sorry to say, originated in a medical journal, and which, by an erroneous statement in regard to the relative proportion of Northern and Southern medical students assembled here, contains a covert appeal to sectional prejudices, against which, as national journalists, we shall at all times raise our voice.

---

The Editor of the Buffalo Medical Journal learns, through a private letter received from Boston, that a movement is in progress to rid the Massachusetts State Medical Society of its homœopathic members. We congratulate the orthodox members of that venerable society, upon having at length waked up to a sense of their duty in this matter. It has always seemed to us passing strange, that our Massachusetts brethren, many of whose names the profession of the country delight to honor, should have consented to sit, year after year, cheek by jowl, with some of the most arrant empirics in Christendom; and it has astonished us none the less, that, under these circumstances, the delegates from this society should have heretofore been permitted quietly to take their seats in the American Medical Association, while the delegates from the Ohio State Medical Society were, on one occasion, sent home for the minor offence of having indorsed the patenting of surgical instruments.

---

We learn from the Boston Med. and Surg. Journal, that the late Dr. Henry Wales, of that city, who died in Paris in June last, has left an extremely valuable collection of books, about fourteen hundred in number, to the library of Harvard College. The collection comprises Sanskrit, German, and Italian literature, of the finest editions and handsomely bound, which were purchased by the testator while residing in Europe. By another clause of his will, Dr. Wales appropriated the sum of forty thousand dollars to be ultimately dedicated to the foundation of a chair of Sanskrit Literature in the University.

---

Notwithstanding the sneering abuse that has been for some time past heaped upon the profession and medical schools of this country by Ranking's Abstract and other British medical journals for what these periodicals are pleased to consider our total lack of a home medical literature, the London University has recently recommended the work of Professor Wood, of this city, on the Practice of Medicine, as a text-book to its students. The compliment is no less highly deserved by Professor Wood, than it is pleasing to his countrymen.

---

Justice is said to be blind, but she is certainly economical in some places, the old Bay State, for instance. A Boston Journal, alluding to the charge against a negro barber in Lowell, of attempting to murder the cook of one of the hotels in that city, by poisoning her drink, says, the Government has dropped the investigation of the matter, for the reason that no analysis of the cider, said to be poisoned, could be had short of fifty dollars.

---

The London Lancet, in a recent notice of the row at the Eclectic Medical Institute of Cincinnati, does not seem to be aware that the college in question is a school of empiricism, in which homœopathy, hydropathy, and all the other fashionable and unfashionable dogmas of the day, are taught, but speaks of it as a scientific body, that has partaken somewhat of the political excitement, "into which the Americans have lashed themselves of late," and winds up with the following serious reflections upon the subject:

"There is a solemn comicality in all this, from its utter opposition to our notions of decorum. It were difficult to imagine a parallel case, unless we could fancy one of our most sedate societies, after a sharp discussion, pouring forth pellmell and 'having it out' in Bowers Street, the president entreating any member just to tread on the tail of his coat, whilst one of the honorary secretaries begged the other to say whether he would like any more. Now the great election is over in America, we opine that a considerable number of the enthusiasts are reasonably ashamed of the antics they have been playing. And we hope that a similar sense of shame has, long ere this, entered the minds of the belligerent professors of Cincinnati."

The foregoing paragraph is in perfect keeping with the general tenor of the Lancet in reference to medical affairs in the United States; and with such informers we do not wonder that the profession of Great Britain generally hold our institutions of learning and the honors they dispense in such low estimation.

---

A scientific expedition, including several eminent gentlemen of this country, is now on its way to South America, for the purpose of making a thorough investigation of the botanical, geological, and mineralogical peculiarities of the countries about the head-waters of the Amazon, following the course of that river to the Atlantic. It is stated that this country has not been traversed by any scientific investigator since the exploration of Humboldt, fifty years ago. The undertaking is strictly a private enterprise, having originated with some gentlemen of Iowa, but whether from purely scientific motives we are not able to say. Professor Silliman, Jr., whose name has been mentioned by the newspapers in connection with the expedition, does not form one of the exploring party.

---

We learn from the Peninsular Journal of Medicine, that the editors and proprietors of that periodical have been ordered to appear in the Circuit Court of the United States for the District of Michigan, to answer to a charge for libel, at the suit of Henry Goadby, who is an alien and subject of the Queen of Great Britain and Ireland, and senior editor of the Medical Independent. The alleged libel was contained in a notice of Dr. Goadby, published in the Sept. No. of the Peninsular Journal.

---

At a late meeting of the Medical Society of London, Mr. Isaac Baker Brown related a case of vesico-vaginal fistula cured in fourteen days by the button-suture. Our friend, Dr. Bozeman, of Montgomery, Ala., the inventor of the operation, an account of which was first given to the profession through the pages of the Louisville Review in May last, will, we imagine, be not a little amused, if not annoyed, to find himself introduced to the profession of Great Britain, by the London Lancet, in which the above case is reported, as Dr. *Bozeneau.* Besides this inexcusable orthographical blunder, the journal in question has copied Dr. Bozeman's plates, and, by omitting to give him the proper credit, ascribes the invention of the instruments therein represented to Mr. Brown. We would beg the editors of the Lancet to explain to us the moral difference between stealing medical books, of which they are in the habit of accusing the publishers of this country, and the theft of which they have been guilty in reference to Dr. Bozeman's operation.

Dr. Spencer Wells, of the Samaritan Hospital, London, is reported in the Medical Times and Gazette as having performed Dr. Bozeman's operation upon a case in which the fistulous orifice was very large, involving the anterior lip of the os uteri. The case had previously been treated frequently and unsuccessfully with the galvanic cautery, and had also been subjected to two operations by other surgeons with similar results. "Up to the eighth day (our last report) all the urine passed by the urethra, and the woman was doing perfectly well."

---

The friends and admirers of Dr. Mütter, late Professor of Surgery in the Jefferson Medical College, will be gratified to learn thathe has in a great measure recovered from the severe attack of gout with which he suffered soon after reaching Paris, and is spending the winter at Nice.

---

At the recent sitting of the Army Medical Board, at St. Louis, only two out of fifteen or twenty candidates succeeded in passing the examination. The fortunate young gentlemen were Drs. Lewis Taylor and S. G. Hollenbush, both of Philadelphia, the former a graduate of the University of Pennsylvania, and the latter of the Medical Department of Pennsylvania College.

---

Professor BLACKMAN recently exhibited to his class at the Medical College of Ohio, a solid ovarian tumor weighing one hundred pounds. The tumor was removed, or rather the patient was removed from the tumor, by Dr. Dunlap, of Ripley, Ohio, and she was reported as doing well on the third day, but we have since learned that the case terminated fatally on the sixth day.

---

A correspondent of the London Medical Times and Gazette, in reply to a question concerning the origin of the phrase Dog Latin, says, that it may possibly be a corruption of "Doctor's Latin," or the Latin employed in medical prescriptions, since the terms used in these documents are sometimes so abbreviated as to justify the application of the above term to language thus *cur-tailed.*

---

A man named Thomas C. Carter, recently died in Tennessee, after having attained the weight of 527 pounds.

---

AMERICAN MEDICAL BIOGRAPHY.—It is known to many of the readers of this journal, that the Senior Editor, more than a year ago, addressed a circular to some of his professional brethren, inviting their aid in the publication of a work of this kind, intended to fill a vacuum, which has long existed in our medical literature. The only work that has ever been issued on this subject, was that of the late Dr. Thatcher, of Plymouth, Massachusetts, well known as the author of a "New Dispensatory," and of a "Modern Practice of Medicine," besides other productions of a minor character. It appeared, in 1828, in two octavo volumes, and was well received by the profession. For a long time this work has been out of print, and there is no probability, as the author himself has gone to that "bourne whence no traveller returns," that another edition will ever be issued. Some years ago, the late Dr. Williams, of Deerfield, Massachusetts, published a volume on American Medical Biography, but it was made up chiefly of selections from the writings of others, and is not at all worthy of a place in our permanent medical literature. What is needed at this time, is an *original* work, embracing an account, brief but graphic, and properly illustrated, of the lives of our most distinguished physicians, surgeons, accoucheurs, and chemists; men who have earned a national reputation, and who, by their conduct and example, have advanced the cause of medicine, science, and humanity. Such a work, we repeat, is much needed, and could not fail to be of vast service to the profession, by showing the world what that profession is, and what it has done. Its circulation would not be confined to medical men, but it would be extensively read by all classes of people, especially clergymen, lawyers, and merchants. In this way might be disseminated a great deal of information, illustrative of the character of the profession, the influence of which would be highly beneficial to the public.

The publication of the work here referred to, has been unavoidably delayed by the removal of the Editor to Philadelphia, and also by the want of punctuality of some of his contributors. He is happy, however, to be able to state, that he has received a sufficient amount of material for the first volume, and that the work will be put to press as soon as material shall be obtained for the second volume. The "Biography" will embrace from thirty to forty memoirs, written by medical gentlemen of acknowledged ability, and will be issued in the highest style of the art, accompanied, wherever this is practicable, by steel engravings. The Editor himself will furnish but one memoir, that, namely, of the late Professor Drake, of Cincinnati.

---

It may not be unknown to most of our readers, that in Vienna a market for the sale of horseflesh has been in successful operation for some time past; the poorer classes being thus supplied, at a comparatively low price, with meat, which is said to be but little inferior to beef. A similar scheme has been lately started in Paris, and by way of giving currency to the new dish, no less a person than M. St. Hilaire, not long since, invited a number of friends to a dinner at which horseflesh, prepared in a variety of ways, was the only meat provided. A large number of savans sat down to the table, and it is said, partook of horse-soup, horse-steak, and roast horse, with all the gusto that the same number of Polynesians would have manifested over a haunch of roasted preacher. It is true that wine formed no inconsiderable part of the feast, but whether this added to the relish of the meat, or vice versa, we are not informed.

In reference to this "latest French touch," the sprightly and amusing writer of the Medical Gossip of the Dublin Medical Press, furnishes us with the following witty *jeu d'esprit:*

"And by the way, talking of the new art of straining at a gnat, a glass of wine with a bee's wing in it, and swallowing—no, not a camel, but a horse; swallowing a horse, 'Hippophagy,' as it is called; it is stated in well-informed circles, that the French having heard of Irish bulls and the mares' nests of the journals, are now not averse to Irish shamrocks and bullocks! and, accordingly, a certain 'capitaliste Anglais' intends to do away with 'Hippophagy' in Paris, of which M. St. Hilaire has written so much good sense, and Dickens and others so much nonsense. He thinks highly of the *betise* of Dublin agricultural produce, and has studied seriously, and has at length elaborated a plan to procure for Paris every week, in place of dead horses, 'du bétail gras d'Irlande.' We now copy textually, as the phrase is, from the *Independence Belge*, the best of the newspapers on this side of the Rhine! Like the Emperor of Russia in *Punch*, who only wants paper security to procure money for his railway, our 'capitaliste Anglais' believes the best category of cows or horses in Paris. 'Sont incontestablement hors de comparison avec le bétail Irlandais;' and if he can only get the Dublin medical and other polyglot journals, in Connemara especially, or at Blarney, where, ahem! Sir Robert Kane has been recently corroborating Dr. Cryan's facts as put forth in the *Times*. If our 'capitaliste Anglais' can only procure a hearing in Dublin and Ballinasloe, 'Hippophagy' is forever ended, and the health of Paris secured forever, by a normal consumption of Kerry cows in place of the horses immortalized with all our oldest 'news' in the pages of a weekly contemporary."

---

NOTICE.—The Editors of this journal wish it to be borne in mind that, excepting personalities, they are not responsible for the sentiments or doctrines enunciated by its various contributors. And it is more especially in reference to the Reviews that they desire to call attention to this fact. Books for review are placed in the hands of such persons in different parts of the country, who are supposed to be able to do them justice, the editors themselves seldom or never engaging in this kind of labor, and no restraint is placed upon a free expression of opinion when this is done in a courteous manner and with a due regard to fact and reason.

